# Role of Honey as a Bifunctional Reducing and Capping/Stabilizing Agent: Application for Silver and Zinc Oxide Nanoparticles

**DOI:** 10.3390/nano13071244

**Published:** 2023-03-31

**Authors:** Norfarina Bahari, Norhashila Hashim, Khalina Abdan, Abdah Md Akim, Bernard Maringgal, Laith Al-Shdifat

**Affiliations:** 1 Department of Biological and Agricultural Engineering, Faculty of Engineering, Universiti Putra Malaysia, Serdang 43400, Selangor, Malaysia; 2 Malaysian Agricultural Research and Development Institute (MARDI), Serdang 43400, Selangor, Malaysia; 3 SMART Farming Technology Research Centre (SFTRC), Faculty of Engineering, Universiti Putra Malaysia, Serdang 43400, Selangor, Malaysia; 4 Institute of Tropical Forestry & Forest Products, Universiti Putra Malaysia, Serdang 43400, Selangor, Malaysia; 5 Department of Biomedical Sciences, Faculty of Medicine and Health Sciences, Universiti Putra Malaysia, Serdang 43400, Selangor, Malaysia; 6 Faculty of Resource Science and Technology, Universiti Malaysia Sarawak, Kota Samarahan 94300, Sarawak, Malaysia; 7 Faculty of Pharmacy, Applied Science Private University, Al Arab St, P.O. Box 166, Amman 11931, Jordan

**Keywords:** nanoparticles, honey, green synthesis, reducing agent, capping agent

## Abstract

The use of natural reducing and capping agents has gained importance as a way to synthesize nanoparticles (NPs) in an environmentally sustainable manner. Increasing numbers of studies have been published on the green synthesis of NPs using natural sources such as bacteria, fungi, and plants. In recent years, the use of honey in the synthesis of metal and metal oxide NPs has become a new and promising area of research. Honey acts as both a stabilizing and reducing agent in the NP synthesis process and serves as a precursor. This review focuses on the use of honey in the synthesis of silver NPs (Ag-NPs) and zinc oxide NPs (ZnO-NPs), emphasizing its role as a reducing and capping agent. Additionally, a comprehensive examination of the bio-based reducing and capping/stabilizing agents used in the honey-mediated biosynthesis mechanism is provided. Finally, the review looks forward to environmentally friendly methods for NP synthesis.

## 1. Introduction

**Figure 1:**
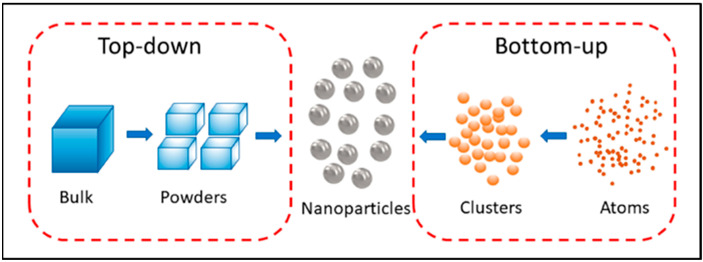
Top-down and bottom-up approaches in the synthesis of NPs [3]. Reprinted from an open-access source.

The synthesis of nanoparticles (NPs) has garnered significant interest due to their diverse applications in various fields, such as biomedicine, food, environment, cosmetics, and electronics. These NPs have demonstrated antimicrobial properties and the capability to catalyze the degradation of organic dyes. In general, NP synthesis can be categorized into two methods: the top-down and bottom-up approaches. The top-down approach primarily employs physical methods and involves converting bulk materials into NPs, while the bottom-up approach uses chemical and biological methods to reduce soluble materials into insoluble NP dispersions [1]. The top-down method usually involves the breaking of Van der Waals forces between stacked components to produce thin crystal layers, whereas the bottom-up approach involves the formation of ionic or covalent bonds [2]. However, Van der Waals forces may also play a role in bottom-up approaches, depending on the specific method used to assemble the material. For example, in the synthesis of NPs, the growth process can involve the aggregation of individual atoms or molecules into larger clusters. Van der Waals forces can play a role in the aggregation process, as the weak intermolecular forces between molecules can help to hold the clusters together. Similarly, in the assembly of molecular structures, Van der Waals forces can contribute to the stability of the assembled structure. Although the primary bonding mechanism, in this case, is covalent bonding, the Van der Waals forces between the individual molecules can also play a role in the overall stability of the structure. The various NP synthesis methods are illustrated in Figure 1.

Recently, efforts have been directed towards optimizing the synthesis of functionalized NPs as carriers for the delivery of antitumor molecules via intravenous or transdermal routes, with the aim of targeting individual cells and minimizing adverse effects associated with systemic administration. Physical methods make use of physical forces to assemble nanoscale particles into massive, stable, and well-defined nanostructures. One example of such a method is the colloidal dispersion approach, which encompasses various fundamental processes such as vapor condensation, amorphous crystallization, physical fragmentation, etc. [4]. In contrast, the chemical synthesis of NPs often involves using various ingredients such as reagents, complexing agents, stabilizers, and surfactants as base chemicals or adjuvants to optimize the properties of the prepared nanophase. The reduction process in the synthesis of NPs requires the utilization of inorganic or organic reducing agents, which can pose potential risks to human health and the environment. Therefore, research is currently focused on developing new economical and eco-friendly reactants to replace hazardous chemicals [5].

**Figure 2:**
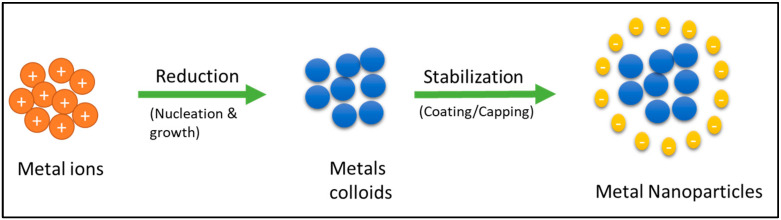
The mechanism of nanoparticle formation.

The bottom-up synthesis of NPs requires the presence of reducing and capping agents for proper control over the final properties of the nanomaterial. According to Indumathy et al. [6], metal colloids are formed through the reduction of metal ions in two stages: nucleation and growth, and the relative rate of these two stages determines the size distribution of the colloids. The concentration of the reducing and capping agents can influence these processes, and the use of capping agents is crucial in ensuring the stability of the NPs by preventing aggregation through the creation of electrostatic, steric, or electrosteric interactions between the particles. The formation of NPs is shown in Figure 2. It is important to note that the interaction between metal precursor ions and reducing agents and the interface between the capping agents and metal NPs can have a significant impact on the morphology and physicochemical properties of the metal NPs [7,8,9].

### 1.1. Reducing Agent

Reductants, also referred to as reducing agents, are compounds that transfer electrons from a solution to ions, resulting in the formation of atoms. The reaction rate during the synthesis process plays a crucial role in determining the size of the formed metal NPs. An excessively fast reaction rate can result in undersized NPs, while a slow reaction rate can lead to NPs aggregation. The choice of surfactant has a significant impact on various properties of the NPs, including stability, solubility, reactivity, dispersibility, size, and shape, during the synthesis process [8].

Excessive amounts of reducing agents are employed in the synthesis of NPs corresponding to stoichiometric amounts. Various chemical agents such as sodium borohydride (NaBH_4_), sodium citrate, ascorbate, elemental hydrogen, *N*,*N*-dimethylformamide (DMF), and polyethylene glycol (PEG) block copolymers are utilized throughout the year to reduce metal ions to zero charged molecules and to form the NPs. This was accompanied by a color shift that becomes darker as the response time is increased [10]. However, the widespread use of these hazardous chemicals has recently been discussed, especially in relation to their impact on human health and safety and the environmental issues associated with their disposal [11]. According to the authors, this complicates the process and makes it difficult to transfer this type of technology to industrial processes.

### 1.2. Capping/Stabilising Agents

A capping or stabilizing agent is a chemical that provides a protective coating to the nanoparticles (NPs) [12]. The use of these agents is essential in NP production to enhance their biological performance by reducing toxicity and improving therapeutic efficacy in biological cells. They avert aggregation of NPs and ensure colloidal stability, thus avoiding unbridled growth of NPs, especially metal and metal oxide NPs [13]. The application of heat treatments, such as calcination or roasting, to NPs in powder form can result in undesired unidirectional mixing. To counteract this, stabilization NPs by anchoring them onto a substrate or enveloping their surfaces with a protective layer of organic ligands (i.e., capping agents) are two commonly employed methods [14,15].

Gulati et al. [16] emphasized that when developing a synthesis method, certain key properties of the surfactant molecule must be taken into account. They reported that the polar head of the surfactant molecule is functionalized with donor atoms such as nitrogen (N), oxygen (O), sulfur (S), and phosphorus (P), which possess lone electron pairs and can form coordinate bonds with metal atoms or ions. The authors further pointed out that the synthesis of NPs frequently involves the utilization of hydrocarbons functionalized with heteroatoms, which are classified based on the character of the donor atom. Other research has emphasized the importance of selecting an appropriate capping agent in NPs synthesis, as it can significantly influence the NPs’ properties with the surrounding medium. Ajitha et al. [7] noted that mechanisms of action, including electrostatic stabilization, steric stabilization, hydration stabilization, depletion stabilization, Van der Waals stabilization, and combinations of these mechanisms, also play a vital role in establishing the type of capping agent to be used for NP synthesis.

### 1.3. Bifunctional Reducing and Capping Agents

In certain scenarios, a particular reagent can act as a bifunctional reducing and capping agent simultaneously. The structure and functional groups of such a dual agent have a significant influence on the reduction process kinetics and, as a result, the final properties of the system. By prudently selecting the reducing agent/capping agent, solvent, and parameters such as temperature and reaction time, the shape and size of the nanomaterials can be manipulated, leading to the creation of NPs with various shapes and sizes, including spherical NPs and anisotropic structures such as plate-like, urchin-like, rod-like, and wire-like, with potential applications in fields such as catalysis, optics, and nanomedicine, among others.

In current materials science research, there is a growing focus on developing novel materials and technologies that can transform traditional procedures by reducing pollution and promoting a clean and sustainable source of energy. One emerging concept in this field is the green chemistry of NPs, which aims to make a practical impact in industrial applications. Muthivhi et al. [10] have identified three key factors to consider in the bio-based synthesis of metal NPs: (i) using a non-toxic capping agent, (ii) using an environmentally friendly reducing agent, and (iii) using an environmentally friendly solvent. To achieve this, a range of approaches to the selection of biomaterials have been explored through various studies. Fourier Transform Infrared (FTIR) analysis has revealed that compounds such as polysaccharides, polyphenols, ascorbic acid, reducing sugars, proteins, and related amino acids play a crucial role in the reduction process, with their functional groups contributing to the reaction. The latter is particularly relevant when by-products from the enzymatic conversion are involved in electron transfer.

In a 2017 review by Vaseghi et al. [17], the function of bioreductants and capping/stabilizing agents in the green synthesis of NPs and their applications were discussed. The authors aimed to survey green sources such as bacteria, fungi, yeast, algae (microbial synthesis), and plants (phytosynthesis) and compare the function of biochemical agents and phytochemical components in the production of green NPs. According to Gusrizal et al. [18], phenolic compounds found in plant metabolites are able to reduce silver ions (Ag^+^) to form Ag-NPs. Hydroxybenzoic acids were found to exhibit antioxidant activity and be suitable for forming Ag-NPs as a reducing and capping agent. The carboxylate group of hydroxybenzoic acid serves as a ligand for coordination with the surface of the Ag-NPs, and the hydroxyl phenolic group acts as a reducing agent. The Ag-NPs coordinated with hydroxybenzoic acid have been shown to have a smaller size and reduced tendency for agglomeration.

The utilization of different plant-derived capping and reducing agents for the production of NPs have been shown to be a promising approach. According to a study by Zahoor et al. [19], the bio-based synthesis of Ag-NPs from plant extracts suggests that secondary metabolites in these extracts can reduce Ag^+^. These secondary metabolites, such as vitamins, polysaccharides, amino acids, proteins, enzymes, polyphenolics, flavonoids, etc., play a crucial role as reducing agents for Ag^+^ and as size control agents in the formulation of Ag-NPs. Moreover, functional groups such as the carboxyl group (COOH) in glutamic and aspartic acid residues and the hydroxyl group (OH) of tyrosine residues have been discovered as key in maintaining the stability of Ag^+^ and the production of small, polydisperse Ag nanoplatelets. This supports the results of Mohamad et al. [20], who reported that the hydroxyl and carboxyl groups present could act as reducing agents and stabilizers in the synthesis of NPs. Conversely, bioactive compounds such as phenolics and alkaloids play a role in capping and stabilizing the produced NPs.

Synthetic chemists and material scientists have expressed interest in finding more environmentally friendly reducing and capping agents to regulate the reduction and stabilization of nanoparticle (NP) dispersions. The need for greener and more scalable production methods of NPs has made the biosynthesis of NPs a viable option for industrial use [21]. The formation of green NPs depends on the oxidation or reduction of metal ions in the biological medium used, as each autotrophy contains different molecules responsible for the process. Despite the specific mechanisms for the green synthesis of NPs not yet being entirely explored [9], it is acknowledged that the synthesis process begins with the nucleation of the metal species and culminates in the formation of NPs as precipitates within the mixture. This review article explores the role of honey as a bifunctional reducing and capping/stabilizing agent and its application in the synthesis of Ag-NPs and ZnO-NPs. Additionally, critical insights and future prospects for its use are highlighted.

## 2. Honey

Honey is a natural food produced by bees through the collection of nectar or flower secretions, and it has been extensively studied to understand its components, physical and chemical properties, and quality control measures. Honey is known for its rich energy content, diverse chemical constituents, and essential enzymes and vitamins, which are believed to support overall longevity. Honey is predominantly made up of carbohydrates, specifically glucose and fructose, which make up approximately 80–85% of its composition. Along with these two sugars, honey also contains various disaccharides and oligosaccharides, such as sucrose, maltose, maltotriose, and panose [22]. Water makes up approximately 15–17%, protein makes up only 0.1–0.4%, and ash makes up 0.2%. Ash content in honey represents minerals and trace elements such as calcium, potassium, sodium, magnesium, phosphorus, sulfur, iron, zinc, copper, and manganese. In addition to these constituents, honey contains small quantities of amino acids, enzymes, vitamins, flavonoids, and phenolic antioxidants [23]. However, the exact chemical composition and physical properties of natural honey can vary depending on factors such as the plant species from which bees collect nectar, climatic conditions, and vegetation [24,25,26,27,28,29,30].

A study conducted by Shamsudin et al. in 2019 examined the physicochemical characteristics, antioxidant properties, and botanical origin differentiation based on organic acid content in Malaysian stingless bee honey [31]. The results revealed that the physicochemical characteristics, organic acid content, and antioxidant properties of the honey were influenced by the bee species and the botanical origin. Furthermore, the study showed that the physicochemical characteristics, organic acid content, and antioxidant properties of stingless bee honey were notably distinct from those of Apis mellifera honey (honeybee honey). Moreover, Rao et al. conducted a study in which they tabulated the physicochemical characteristics of various types of honey, including honey bee (Tualang and Manuka honey) and stingless bee honey [22]. A similar study was also conducted by Kamal et al. [32]. However, in their study, they substituted the Manuka honey with Gelam honey. Based on their analysis, both studies concluded that the different types of honey exhibit diverse biological and therapeutic effects. 

On top of that, recently, Haron et al. [33] conducted a study to assess the inherent differences in the chemical composition of stingless bee honey collected from the east coast, central, and northern regions of Peninsular Malaysia. The findings showed that the honey collected from the east coast region had the highest levels of total flavonoids and phenolics. However, the levels of proline and ascorbic acid appeared to be less influenced by geographical factors. Additionally, acid taste, acid flavor, honey flavor, and sweet taste were also identified as important factors. These findings indicate that the floral source of honey affects not only its color but also its acidity, sugar profile, ash content, and proline content [34,35].

### Honey as Bifunctional Reducing and Capping/Stabilising Agent

The synthesis of nanoparticles (NPs) can be initiated by various compounds present in plant extracts and microbial cells, such as carbonyl groups, terpenoids, phenols, flavonoids, amines, amides, proteins, pigments, alkaloids, and other reducing agents [36]. Despite its exceptional qualities, honey has not yet been thoroughly researched by scientists worldwide. Honey possesses unique chemical properties that make it a valuable tool for the green synthesis of NPs. Honey-based synthesis offers several advantages over traditional microbial approaches and plant-based methods. Notably, honey-based synthesis is a rapid system compared to microbial approaches, which contributes to its effectiveness as a green synthesis method. Several researchers reported that glucose and fructose in honey are the primary reducing agents. These sugars are capable of reacting with different reagents, including Benedict’s (Cu^2+^ to Cu_2_O), Tollens’ (reduction in Ag^+^ to Ag^0^), and Fehling’s [37]. Additionally, its glucose and fructose content makes it a potential candidate for use in the green synthesis of NPs as a capping and reducing agent [38]. Honey is also notable for its low pH (acidic) and hydrogen peroxide compounds, and it has been found that it can convert silver ions into Ag-NPs at alkaline pH, which is surprising [39].

**Figure 3:**
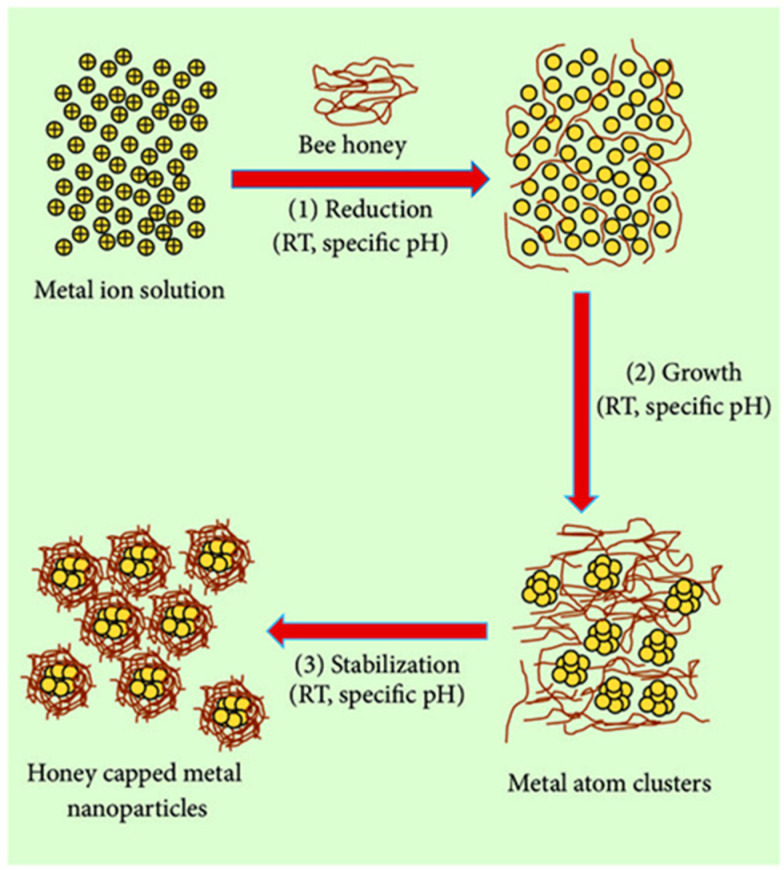
Schematic diagram of honey-mediated green synthesis of NPs [24]. Reprinted from an open-access source.

Recently, the concept of honey-mediated green synthesis has gained traction as a method of synthesizing gold NPs (Au-NPs) [40,41], Ag-NPs [36,42,43], carbon NPs [44], platinum NPs (Pt-NPs) [45], and palladium NPs (Pd-NPs) [46]. Typically, honey functions as both a reducing agent and a capping/stabilizing agent in the NP synthesis process, primarily serving as a precursor [47]. The preparation of NPs using honey follows a general mechanism that involves the following steps [48]: Initially, the metal salt solution undergoes dissociation to produce positively charged metal ions (Me^n+^). Then, these metal ions react with the negatively charged hydroxyl (OH) groups of fructose and glucose present in the honey to form the complex [Me(honey)]^n+^. The addition of sodium hydroxide (NaOH) triggers a reaction that results in the formation of the complex ([Me(OH)_n_/honey]). Finally, through continuous stirring and aging, a metal or metal oxide NP is formed (Figure 3).

The use of honey as a sole precursor in the synthesis of NPs has shown similar results to those obtained from the use of glucose or fructose, as it demonstrates a comparable trend in terms of particle size reduction during NP production. Particle size decreases with increasing concentration of glucose. The reducing properties of fructose, glucose, and vitamin C in honey facilitate the formation of metal NPs by inducing the oxidation of glucose in an alkaline environment with the addition of NaOH, resulting in the formation of gluconic acid [24]. 

Besides fructose and glucose, proteins and/or enzymes may also play a role in this oxidation process. For example, glucose oxidase is an enzyme found in honey that catalyzes the oxidation of glucose to gluconic acid and hydrogen peroxide. This process can lead to the formation of metal oxides in contact with the metal NPs. Similarly, catalase and peroxidase can catalyze the breakdown of hydrogen peroxide, which can also lead to the formation of metal oxides. On the other hand, proteins in honey can also interact with metal NPs and influence their properties. Proteins in honey can act as capping agents, surrounding the NPs and preventing them from agglomerating or oxidizing [43]. Additionally, proteins can influence the size, shape, and stability of the nanoparticles by controlling the rate of nucleation and growth.

In addition, the presence of phenolic acids in honey can also contribute to NP formation through synergistic effects. Phenolic acids have been reported to act as effective reducing agents due to their multiple hydroxyl groups and as efficient dispersants due to their derived C=O groups [49,50]. These groups make phenolic acids effective dispersants, which means that they are capable of preventing NPs from clumping together and aggregating. When NPs are well dispersed, they are more stable and less likely to undergo aggregation or precipitation. This means that the combination of reducing and dispersing properties of phenolic acids can work together to enhance NP stability.

Similar to other biosynthesis methods for NPs, the exact mechanisms behind the reduction and stabilization of NPs produced using honey have yet to be fully understood. Fourier-Transform Infrared (FTIR) spectroscopy is a useful device for determining the biomolecular composition of samples before and after the reaction. The FTIR spectra can provide insights into the bioactive molecules that are crucial in reducing and maintaining the stability of the NPs. Generally, FTIR peaks correspond to the vibrations of the functional groups present in a sample. When comparing the FTIR spectrum of metal or metal oxide NPs synthesized with honey to the FTIR spectrum of honey extracts, several distinctive FTIR peaks of the honey may vanish, become less intense, or shift, which could suggest a reduction process. Conversely, if the FTIR peaks remain unchanged, it suggests that the functional groups responsible for stabilizing the NPs are unchanged.

A study conducted by Philip in 2009 [40] was among the initial investigations into the green synthesis of Au-NPs with honey. In his study, the author reported that when excess honey was used to reduce aqueous chloroauric acid (HAuCl_4_), the capping agent biomolecules formed strongly spherical NPs and not nanotriangles, although the reductive biomolecules were enhanced. The author also found that smaller amounts of honey satisfied the reduction of chloroaurate ions but did not protect most of the quasi-spherical NPs from aggregation due to the lack of biomolecules as protective agents. These studies agree with those of Maringgal et al. [51] for the biosynthesis of calcium oxide NPs (CaO-NPs) calcined for 1 h. FTIR spectrums revealed the presence of hydroxyl groups, carboxyl groups, amines, and amides. This confirms the possibility of the phytochemical properties of *Trigona* sp. secretions towards improving NP stability. Therefore, the author concluded that the proteins present in honey could bind to metal NPs via free amine groups or carboxyl ions of the amino acid residues contained therein. 

Back in 2010, Philip repeated his synthesis of NPs utilizing honey as source material. During this iteration, the synthesized NPs were identified as Ag-NPs [42]. In this study, the addition of NaOH causes a reduction in Ag^+^. The alkali promotes the opening of the glucose ring by extracting α-protons from the oxygen of the sugar ring, and the metal ion oxidizes glucose to gluconic acid. From the results, he concluded that glucose, sucrose, and proteins/enzymes also play a role in reduction and, at the same time, be involved in stabilizing Ag-NP as a capping material. In addition, the honey extract has a more negatively charged functional group in an alkaline environment and is capable of fully binding and reducing silver nitrate (AgNO_3_) to Ag-NPs. However, under acidic conditions with aggregation of bacterial formation, it promotes particle dispersion [50]. At the same time, it has been reported that high temperatures promote the nucleation process, and low temperatures promote growth in the field of wet chemical NPs synthesis. Increasing the reaction temperature reduces the particle size, allowing larger NPs to be obtained at relatively lower temperatures.

Meanwhile, in 2019, Ismail et al. [52] postulated a promising mechanism for the production of copper nanoparticles (Cu-NPs) using honey. During the reaction, copper ions (Cu^2+^) from copper nitrate react with the negative charge on the hydroxyl group of fructose and glucose in honey to form [Cu(Honey)]^2+^. Upon the addition of NaOH, [Cu(OH)_2_/honey] is formed, and Cu-NPs are produced after the addition of ascorbic acid and ultrasonic irradiation. This shortens the reaction time. The positive charge on the surface of the Cu-NPs forms an electrostatic bond with the negative charge of the OH group in fructose and glucose of honey, resulting in a uniform particle size distribution. Meanwhile, the polyhydroxy groups of glucose and fructose prevent large agglomerates from forming. On the other hand, Yousaf et al. [53] found that the OH ions play an important role in the structural changes in zirconia coatings. The hydrophobic nature of honey prevents water uptake in the zirconia lattice, thus helping to stabilize the tetragonal zirconia phase. However, from the study, they discovered that the evaporation of an organic species takes place before the reaction mechanism is complete, thus, leading to the presence of mixed monoclinic, tetragonal phases.

In addition, studies of the impact of honey concentration on the synthesis of NPs have also been performed. Czernel et al. [54] conducted a study to synthesize Ag-NPs using aqueous honey solutions at 2%, 10%, and 20% concentrations. The reactions were performed at 35 °C and 70 °C. The presence and properties of the Ag-NPs were confirmed through ultraviolet-visible (UV-Vis) and fluorescence spectroscopy, scanning electron microscopy (SEM), and dynamic light scattering (DLS) analysis. The results showed that the 20% honey solution inhibited the synthesis of NPs at 35 °C, and this inhibition was found to be related to protein aggregation, as proved by resonance light scattering (RLS) instrumentation.

Additionally, the impact of varying honey concentrations at a constant pH on the shape of the produced Ag-NPs has been previously documented by Haiza et al. [43]. The results showed that honey concentration influences the particle size of the Ag-NPs. In an aqueous solution, a higher concentration of honey decreases the size of Ag^+^ ions compared to a lower concentration. This is attributed to the presence of components in honey such as fructose, glucose, various acids, vitamins, and minerals that act as reducing and capping agents. Moreover, other components such as sucrose and proteins/enzymes in honey may also contribute to the degradation of Ag^+^ ions. This is consistent with the findings by Rasouli et al. [55], who performed a green and simple synthesis of superparamagnetic iron oxide NPs (Fe_3_O_4_-NPs) and controlled the particle size of the NPs by changing the concentration of honey.

On top of that, Rasouli et al. [55] also conducted a comparison of NPs size between those produced using honey and those produced through synthetic methods. Their findings revealed that the NPs generated from honey were relatively smaller in size when compared to those produced using other methods. Similar results have been presented by Ismail et al. [52], in which they reported that the use of honey in the synthesis of Cu-NPs led to the formation of smaller particles compared to those generated without the presence of honey. This is due to proteins and monosaccharides in honey that act as stabilizing agents, preventing the NPs from agglomeration and, at the same time, narrower distribution of particle size for the generated NPs. Furthermore, they also uncovered that the shape of Cu-NPs produced without honey exhibited a greater degree of inconsistency when compared to those synthesized using honey.

Overall, the use of honey as a reducing and capping agent in the synthesis of nanoparticles (NPs) has been demonstrated, although results among researchers are inconsistent. Table 1 summarizes the reducing agents and capping/stabilizing agents used in honey-mediated NP synthesis and their applications. The variability in results is likely due to multiple factors, and further research is needed in this area. Moreover, there is a need to evaluate natural substances for various applications with minimal adverse effects or risks of overdose or overconsumption. Recently, there has been a significant amount of research on the use of honey to produce Ag-NPs and ZnO-NPs. These NPs are typically stable, hydrophilic, and have small diameters.

## 3. Silver and Zinc Oxide Nanoparticles

The versatility of Ag-NPs and zinc oxide NPs (ZnO-NPs) in rendering themselves to many applications, including in sensors [58], renewable energies [59], environmental remediation [60], bio-therapeutic devices [61], clothing [62], antimicrobial [63] is currently being explored. This is due to the unique properties of their small size and high surface area-to-volume ratio, which enhances their physical, biological, and chemical characteristics compared to those of their bulk counterparts.

Ag-NPs are considered noble metal NPs due to their favorable properties, such as good tolerability and targeted interactions, and are commonly used as delivery vehicles for cellular compounds. Ag-NPs also exhibit potent antibacterial activity [64]. ZnO-NPs, modified from bulk ZnO, have been used for their strong antibacterial and anti-inflammatory effects [65,66]. Kyomuhimbo et al. [67] demonstrated the synergistic effect of Ag and ZnO in increasing the antibacterial activity of a nanocomposite by producing reactive oxygen species (ROS), such as hydrogen peroxide (H_2_O_2_). The study found that Ag-NPs and ZnO-NPs exhibit potent antibacterial activity at low concentrations, even against drug-resistant bacterial strains. The NPs disrupt multiple biological pathways in a cell and would require simultaneous mutations for the microorganism to develop resistance, making them effective even with short exposure times.

According to Singh et al. [68], Ag and ZnO have an extensive history of use as antimicrobial agents and are currently being used as powerful antibacterial agents in wound dressings. The antibacterial activity of Ag-NPs and ZnO-NPs is dependent on their physical properties, such as size and shape. The study showed that smaller NPs have a larger surface area, leading to an increased binding affinity between molecules. Ag-NPs and ZnO-NPs have also been shown to be biocompatible in human cells, making them an active area of research. This finding is consistent with a study by Jafarirad et al. [69], which showed that synthesized Ag/ZnO nanocomposites (Ag/ZnO-NCs) exhibit antioxidant activity while causing no significant harm to human A549 cells.

Apart from that, Hu et al. [65] emphasized that both Ag and ZnO at lower concentrations inhibit but do not kill microbes. It is believed that they allow certain microbial cells to survive and hence build resistance to the antibiotic when exposed to it. As a result, a dependable drug delivery system that can preserve the anti-infection superiority of Ag-NPs and ZnO-NPs while decreasing the medication administration dose without compromising their ability to kill bacteria is very desirable. Despite efforts to develop ZnO or Ag formulations, ideal antibacterial efficacy, and low toxicity remain challenges. Ag materials, particularly Ag ions, on the other hand, can boost the antibacterial activity of ZnO. Both ZnO and Ag concentrations could be lowered without compromising therapeutic efficacy. As a result, this combination of ZnO/Ag bimetallic nanoparticles will be the most effective solution to overcome the limits of metal compounds in antimicrobial treatments.

### 3.1. Honey-Mediated Silver Nanoparticles

The synthesis of Ag-NPs using honey typically involves the addition of a silver salt precursor, such as AgNO_3_, to a honey solution. Table 2 summarizes the various silver salt precursors used for Ag-NPs synthesis, conditions, morphologies of the resulting Ag-NPs, and their applications. According to a study by Khorrami et al. [70], the appearance of a brownish-yellow color followed by a dark brown color after a few hours of contact confirms the formation of Ag-NPs through the suggested reaction.

Ag^+^NO_3_^−^ + Honey (OH, COOH, etc.) → Ag^0^ NPs (1)

In 2017, González Fá et al. [71] drew special attention by suggesting the synthesis of Ag-NPs using honey under acid and alkaline conditions at room temperature. According to those authors, the synthesis of Ag-NPs is accelerated in alkaline media. The presence of OH groups from honey reacts with Ag^+^ to form Ag_2_O and then forms the Ag(OH)_x_ complex. Eventually, Ag^0^ is obtained, which increases the nucleation rate. The OH groups in the solution also open the glucose ring. The rate of reaction increases with honey concentration. The effect as a reducing agent, in this case, shows that a higher concentration of the reactant increases the rate of the reaction. However, with a high concentration of honey, the concentration of OH groups can be reduced due to the buffering capacity of honey. This can inhibit the formation of intermediate complexes and decrease the reduction and nucleation of silver ions. To summarize, the mechanism described above transforms Ag^+^ ions to Ag^0^ particles through the linear form of glucose. Then, glucose molecules are adsorbed on the reduced silver, acting as capping agents.

It has been suggested that the reduction process of Ag^+^ ions to Ag-NPs could also involve sucrose, glucose, and proteins/enzymes. The addition of NaOH to the solution can impact the size of the NPs formed as it increases the pH of the solution, leading to the production of more gluconic acid from glucose. This occurs because the alkali abstracts the α-proton from the sugar ring of glucose and causes the opening of its ring structure, leading to the formation of gluconic acid. The Ag^+^ is reduced to metallic Ag^0^ after the oxidation of glucose to gluconic acid [43]. In a recent study, Ghramh et al. [72] reported that AgNO_3_ was mixed with the diluted form of honey to prepare Ag-NPs. The change in mixed color was evidence of the formation of Ag-NPs. Sugars (sucrose and glucose) and proteins (possibly enzymes) play a role in the Ag^+^ reduction process. In addition, the addition of NaOH, which increases the pH of the solution, had a significant impact on the expected volume of produced NPs. FTIR spectroscopy analysis revealed the existence of multiple functional groups in honey as evidence for the source of reducing and capping agents.

Notably, both findings by Haiza et al. [43] and Ghramh et al. [72] are parallel to those of Hemmati et al. [73]. In the paper, the authors claim that reducing sugar is sugar that can serve as a reducing agent and contains either free aldehyde or free ketone groups. They denote that all monosaccharides (simple sugars) are reducing sugars, while sucrose (table sugar) and maltose are non-reducing sugars. According to the authors, when heated in an alkaline state, reducing sugars are generally converted to gluconate and other compounds that can serve as reducing agents. In addition, alkaline hydrolysis has been proven to convert non-reducing sugars such as sucrose into reducing sugars such as glucose and fructose, which are responsible for the reduction of Ag^+^ ions to Ag^0^ atoms. Furthermore, the pH value and amount of the alkaline solution employed during the synthesis operations can have a substantial influence on the efficiency of Ag-NP formation [74]. 

The study by Czernel et al. [54] supports previous findings that the reduction of glucose and fructose in honey is the key factor in the synthesis of Ag-NPs. They found that proteins act as stabilizing agents while an alkaline environment is necessary for synthesis. The researchers observed that at high pH, the removal of a proton from the oxygen ring of glucose facilitated the opening of its ring, leading to the oxidation of metal ions into gluconic acid. The formation of NPs was characterized by a change in the color of the synthesis mixture from pale yellow to light brown. This change was attributed to the surface plasmon resonance (SPR) of the Ag-NPs, as evidenced by its corresponding UV-Vis spectrum.

FTIR measurement was performed to identify the bioactive molecules responsible for the capping and stabilization of Ag-NPs synthesized using honey. According to the study conducted by Al-Zaban et al. [39], FTIR detected the presence of phenolic compounds through a broad and prominent peak at 3350.87 cm^−1^, which was due to the hydrogen-bonded O-H stretching. The stretching of the aromatic C-H bond produced peak bands at 2957.76 and 2882.84 cm^−1^. The strong peak at 1634.25 cm^−1^ was attributed to the C-C stretchable alkene functional group. The bands at 1418.21 cm^−1^ were assigned to the N-H stretching vibration of proteins. Furthermore, the peaks associated with the C-O single bond at 1248.41 cm^−1^ and 1044.16 cm^−1^ may correspond to the vibrational frequencies of amide proteins, with frequencies ranging from 1000 cm^−1^ to 1300 cm^−1^ depending on the type of molecule.

The results are consistent with those reported in the 2020 study by Al-Brahim and Mohammed [35], where Ag-NPs were synthesized from *Z. spinachristi* sp. and *A. gerrardii* sp. honey. FTIR detected the presence of phenols or glycosides as stretching vibrations of H-bonded OH groups. Another study led by Ghramh et al. [26] found different peaks in FTIR spectra for different types of honey. The reduction of AgNO_3_ to Ag-NPs resulted in the formation of several compounds, with the disappearance of peaks belonging to cyclopentanone, alkene, and bromo compounds, indicating their involvement in the reduction and stabilization of the Ag-NPs.

Sreelakshmi and colleagues [75] reported that the high dispersion levels of the NPs were attributed to the intrinsic properties of the metals, including surface energy and melting point. Additionally, the transmission electron microscopy (TEM) results indicated that no agglomeration occurred, which suggested that the functional groups in the honey played a critical role in firmly anchoring the formation of the Ag-NPs. Furthermore, the co-existence of other elements with silver in honey corresponds to its functional properties, and these entities are proposed to bind efficiently to Ag-NPs, thereby preventing them from agglomerating.

The exact mechanism for the green synthesis of Ag-NPs using honey is not well understood. However, researchers concur that biomolecules such as reducing sugars, proteins, phenols, and flavonoids in honey play a crucial role in reducing metal ions and capping the Ag-NPs. The current authors deduce that the free amino groups and/or carboxyl residues of honey bee biomolecules, which could be proteins or polysaccharides, bind to Ag-NPs. These biomolecules are not only responsible for reducing Ag^+^ to Ag^o^, but also act as stabilizing capping agents for Ag^o^, potentially enhancing the biological properties of the produced Ag-NPs. It has been established that amino groups are primarily the main agents in the reduction of Ag^+^ ions and are adsorbed by them to a considerable extent.

### 3.2. Honey-Mediated Zinc Oxide Nanoparticles

The stability of zinc oxide ZnO-NPs is influenced by the choice of capping agents utilized in the synthesis process. These agents play a significant role in guiding the formation of diverse ZnO nanostructures. Understanding the particle development process is important for controlling the shape of ZnO-NPs. Honey contains a range of carbohydrates, enzymes, vitamins, OH groups, and amine groups, which can help to complex zinc cations (Zn^2+^) into the initial molecular matrix. This structure enables zinc species, eventually forming ZnO-NPs, to be stabilized and coated, preventing excessive aggregation and crystal formation. This method provides a simple, environmentally friendly, and economically feasible way to produce various nanopowders [76,77]. A summary of the zinc salt/precursor used, synthesis conditions, ZnO-NPs morphology, and applications are presented in Table 3. The reaction between zinc nitrate solution and honey generally leads to the formation of ZnO-NPs.

(NO_3_)_2_^−^Zn^2+^·6H_2_O + Honey (OH, COOH, etc.) → ZnO NPs (2)

Basnet Chatterjee [14] conducted experiments using in-situ grazing incidence diffraction (GID) to study the particle growth kinetics and sedimentation near the substrate surface. They found that ZnO with a crystalline hexagonal phase could form both in the solution phase and in the dried state. They determined the critical concentration required to fully understand the growth kinetics of ZnO-NPs and observed that ZnO particles nucleated in solution and increased in size over time. The growth kinetics were detected at the critical concentration and continued during the sedimentation process. Capping agents can be used to inhibit the growth of certain facets and produce ZnO-NPs with a specific morphology.

In a study by Jeyageetha et al. [79], ZnO-NPs were synthesized using honey as a medium and characterized using various techniques. The results showed that the FTIR peaks of metals and their oxides appeared at lower wavenumbers between 400 to 800 cm^−1^. The study also noted absorption peaks at various wavenumbers that were attributed to specific molecular bonds such as O-H stretching, aliphatic asymmetric C-H stretching, O-H stretching in carboxylic acid, NH bending of amines, C-C stretch of aromatics, -C=N stretches, amide bands of proteins, O-H stretching vibrations of carboxylic acids, tetrahedral coordination of the Zn ion, and C-H bending of aromatics. 

Recently, UV-Vis spectra were acquired in the range of 200 to 800 nm to confirm the existence of ZnO-NPs. A change in color from yellow to brown during the synthesis process was observed, which indicated the reduction of Zn^2+^ ions to ZnO-NPs by electron transfer. FTIR analysis was employed to identify the biomolecules that contributed to capping and stabilizing the synthesized sample. Two absorption peaks at 1449 cm^−1^ and 1124 cm^−1^ were found in the spectra and corresponded to the protein conformations of the capped ZnO-NPs and vibrations of C-O and C-N, respectively. The release of zinc and oxygen from bare zinc oxide was also observed, which indicated the existence of ZnO-NPs on the surface of the nanoparticles and was shown by an absorption peak at 515 cm^−1^. These findings indicate that the stabilization of ZnO-NPs is formed by the interaction between carboxyl ions and amino acid groups. Other weak peaks are also attributed to the interaction of ZnO-NPs with amino acid residues. It was concluded that components of honey, such as fructose, glucose, sucrose, proteins, minerals, and vitamins, may play a role in the synthesis of ZnO-NPs [78]. 

Ranjithkumar et al. [81] conducted a study to compare the impact of using honey and cow urine on the properties of ZnO-NPs synthesized through the co-precipitation method, including the crystalline size, structure, morphology, band gap, and thermal properties. The results showed that the hexagonal structure at pH 8 transformed into stable nanospheres at pH 12, with honey having a noticeable impact on the morphological change of ZnO-NPs. Another study by the authors found that honey-assisted ZnO-NPs exhibited high zeta potential, high thermal stability, and strong antibacterial activity against both Gram-positive (*B. subtilis*) and Gram-negative (*E. coli*) bacteria [80]. Furthermore, the results indicated that the ZnO-NPs synthesized with honey had better colloidal stability and more uniform particle dispersion compared to those synthesized with cow urine.

To date, limited research has been conducted on synthesizing ZnO nanoparticles (NPs) using honey, and further investigation is required to determine the specific substances responsible for metal ion reduction. While proteins have been suggested to play a role in stabilizing ZnO-NPs in some experiments, further study is necessary to identify the specific proteins involved in functionalizing these NPs.

## 4. Conclusions and Future Perspectives

The increasing focus on green chemistry and biological processes has created a need for environmentally friendly methods of NP synthesis. The properties of metal and metal oxide NPs, such as size, shape, composition, and structure, play a crucial role in determining their characteristics. The development of high-quality NPs with controlled physical and chemical properties is essential. Honey-functionalized NPs possess unique properties, such as catalytic activity, anticorrosive activity, antibacterial activity, and potential for use in biosensing and bioimaging. With further research, honey-mediated green NPs may provide a more environmentally friendly alternative to traditional methods and have various applications in various fields.

The utilization of honey in the synthesis of NPs has been found to have both reducing and capping properties, which leads to improved size and shape stability and increased yield. Although the exact mechanisms involved in the biosynthesis of honey-mediated NPs remain unclear, it is believed to start with the nucleation of the metal ions followed by the growth of NPs as a precipitate in the solution. The organic components in honey also contribute to the stability of the NPs, as they bind to the surface and prevent aggregation, eliminating the need for additional stabilizing agents. However, more research is needed to fully understand the reaction steps and specific compounds responsible for the synthesis of these NPs.

There is potential for scaling up laboratory-based studies of honey-mediated NP synthesis to an industrial level, identifying the phytochemicals involved in NP synthesis, and determining the precise mechanism for NP quality development. Honey-based NPs have a wide range of applications in food, pharmaceutical, and cosmetic fields, making them a valuable area of study. Other experimental parameters, such as pH, incubation temperature, and precursor concentration, should also be studied to identify optimal conditions for using biomaterials to obtain well-dispersed and stable NPs. The pH of the medium is particularly important in controlling reaction yield, as it can greatly impact the role of active sites as electron donors and influence both the reduction activity and the capping process. For example, changing the pH can alter the shape of NPs, and increasing pH, reaction temperature, and extract concentration has been shown to decrease the average size of NPs.

## Figures and Tables

**Table 1:** Reducing agents and capping/stabilizing agents used in honey-mediated NPs and their applications.

Type of NPs	Reducing Agent	Capping/Stabilizing Agent	Size of NPs Formed (nm)	Application of NPs	References
Ag	Glucose, Fructose	Protein	4.18 to 18.17	Antimicrobial	[38]
	Glucose, Fructose, Vitamin C	Glucose, Fructose, Protein/Enzymes	5 to 25	Catalytic degradation of methylene blue	[39]
	Glucose	Protein	4	Na	[42]
	Amide and amine groups, phenolic compounds, carbonyl groups	Protein	100	Antimicrobial	[50]
	Glucose, Fructose	Protein	42 to 80	Antifungal	[54]
	Fructose, Glucose, Sucrose, Protein/Enzyme, Vitamins, Minerals, Organic acids	Fructose, Glucose, Sucrose, Protein/Enzyme, Vitamins, Minerals, Organic acids	15.63 to 26.05	Na	[43]
Cr_2_O_3_	Carbohydrate	Phenolic compounds	24	Antioxidant and antibacterial	[47]
Au	Fructose	Protein	15	Na	[40]
	Fructose, Vitamin C	Protein	14.1 to 14.5	Antioxidant and Catalytic activity	[56]
	Ns	Ns	20 to 50	Na	[57]
CaO	Phytochemical compounds	Protein	100	Antifungal	[51]
Cu	Glucose, Fructose, Protein, Ascorbic acid	Protein, Glucose, Fructose	3.68	Antibacterial	[52]
Tetragonal ZrO_2_	Ns	Ns	17.44 to 23.01	Optical	[53]
Fe_3_O_4_	Fructose	Protein	2.22 to 3.21	Na	[55]

Na—not applicable; Ns—no specific compounds mentioned.

**Table 2:** Silver salt/precursor used, synthesis condition, Ag-NPs morphology, and their applications.

Silver Salt/Precursor	Synthesis Condition	Shape	Size (nm)	Application	References
AgNO_3_	- Reaction temperature, 30 °C. - Incubation time, 72 h in dark conditions.	Spherical	50 to 98	Antioxidant, antibacterial	[35]
AgNO_3_	- Reaction temperature, 35 °C - Stirred vigorously for 24 h.	Spherical	42.7	Antibacterial	[70]
AgNO_3_	- centrifuged at 12,000 rpm for 1 h. - ph mixture at 5 to 10.	Spherical	20.0	Na	[71]
AgNO_3_	- pH mixture at 6.5 to 8.5.	-	15.63 to 26.05	Na	[43]
AgNO_3_	- Continuous stirring until color changes to brown under room temperature conditions.	Spherical	50 to 90	Anticancer, antimicrobial, immunomodulatory	[26]
AgNO_3_	- Continuous stirring until color changes to brown under room temperature conditions.	Spherical	60 to 85	Antimicrobial, immunomodulatory	[72]
AgNO_3_	- pH mixture was adjusted to 9.5	Spherical	42 to 80	Antifungal	[54]
AgNO_3_	- stirred for 1 min. - pH mixture was adjusted to 6.5.	Spherical	5 to 25	Catalytic degradation of methylene blue	[39]

Na—not applicable.

**Table 3:** Zinc salt/precursor used, synthesis condition, ZnO-NPs morphology, and their applications.

Zinc Salt/Precursor	Synthesis Condition	Shape	Size (nm)	Application	References
Zn(NO_3_)_2_·6H_2_O	- Reaction temperature, 60 °C. - Stirred continuously for 1 h. - Drying at 100 °C for 1 h. - Annealed 550 °C, 2 h.	Quasi-Spherical	39	Photocatalytic degradation of methylene blue, antibacterial, antifungal	[78]
Zn(NO_3_)_2_·6H_2_O	- Reaction temperature, 60 °C in oil bath. - Incubated for 6 h. - Annealed 200, 400, 600, and 800 °C for 2 h.	Spherical	30	Na	[76]
Zn(NO_3_)_2_·6H_2_O	- Continuous stirred at 60 °C until paste formed. - Annealed 400 °C for 2 h.	Plate-like and rod-like structure	26	Na	[79]
Zn(NO_3_)_2_·6H_2_O	- Stirred for 1 h. - Heating at 100 °C with continuous stirring until powder obtained.	Spherical	23	Antimicrobial	[80]

Na—not applicable.

## Data Availability

Not applicable.
